# Peroxidase Gene *CaPOD49* Suppresses Chilli Veinal Mottle Virus Infection and Increases Oxidative Stress Tolerance in Chilli Pepper

**DOI:** 10.1111/mpp.70222

**Published:** 2026-02-13

**Authors:** Guangqi Wang, Juanjuan Xu, Pingchuan Zhu, Zheng Cai, Youzhi Li, Mingxia Gong, Bihong Feng, Risheng Wang, Xianwei Fan

**Affiliations:** ^1^ State Key Laboratory for Conservation and Utilization of Subtropical Agro‐Bioresources, College of Life Science and Technology Guangxi University Nanning Guangxi China; ^2^ Institute of Vegetable Research Guangxi Academy of Agricultural Sciences Nanning Guangxi China; ^3^ College of Agriculture Guangxi University Nanning Guangxi China

**Keywords:** *Capsicum annuum*, *Chilli veinal mottle virus*, gene silencing

## Abstract

Chilli veinal mottle virus (ChiVMV) is the most important virus known to threaten chilli pepper ( *Capsicum annuum* ) growth and yield in Asia and Africa. Here, we identified the class III peroxidase gene *CaPOD49* as a key regulator of chilli pepper immunity against ChiVMV. The *CaPOD49* expression was strongly induced in response to ChiVMV infection at 48 h post‐inoculation in both resistant and susceptible pepper cultivars. Silencing *CaPOD49* via virus‐induced gene silencing (VIGS) significantly increased disease severity, reduced plant survival rates and increased the disease index of ChiVMV infection. This susceptibility was associated with the elevated accumulation of reactive oxygen species (ROS), while the treatment with the ROS scavenger diphenyleneiodonium (DPI) recovered pepper resistance. Additionally, the defence‐related genes *CaPR2*, *CaPR5* and *CaPR10* were significantly suppressed in *CaPOD49*‐silenced plants, as well as increasing its lipid peroxidation level. Heterologous expression of *CaPOD49* in yeast enhanced its oxidative stress tolerance, consistent with the protective effects demonstrated by its peroxidase activity in vitro assays. Our findings demonstrate that *CaPOD49* positively regulates chilli pepper immunity by fine‐tuning ROS levels and activating defence responses, offering potential genetic targets for disease‐resistant chilli pepper breeding.

## Graphical abstract

*CaPOD49*, a plasma membrane‐localized class III peroxidase, positively regulates chilli pepper immunity against ChiVMV by scavenging RBOH‐generated ROS to maintain redox homeostasis and activate defense gene expression. VIGS‐mediated silencing of *CaPOD49* causes excessive ROS accumulation, suppression of defense signaling, and enhanced viral susceptibility, which can be rescued by the NADPH oxidase inhibitor DPI.

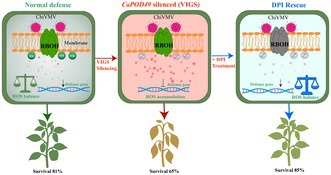

## 1 Introduction

Pepper ( *Capsicum annuum* ) is one of the most important vegetable crops grown in China, which can be used for multiple purposes, such as food and medicine. It suffers from diverse pathogen infections. Chilli veinal mottle virus (ChiVMV) is one of the most destructive viral diseases for pepper growth and production, and has caused a 30%–50% yield loss in Asian countries depending on the infection severity (Lee et al. 2017; Aleem et al. 2022). The breeding of resistant cultivars to ChiVMV infection is essential for the development of the pepper industry.

Several genes are known to be involved in restricting viral spread of ChiVMV in pepper. For example, the silencing of *cvr4* (DEM.v1.00021323) can generate complete resistance to ChiVMV infection in a susceptible cultivar (Lee et al. 2025). *pvr1* ^*2*^ and *pvr6* genes are mutants of *eIF4E* and *eIFiso4E*, respectively; in order for ChiVMV infection to occur, the viral genome‐linkage protein (VPg) must interact with eIF4E and eIFiso4E; however, the *pvr1^2^* and *pvr6* mutants prevent the ChiVMV infection in pepper (Hwang et al. 2009). The ChiVMV helper component proteinase promotes viral infection by directly interacting with catalases in tobacco plants (Yang, Yuan, et al. 2020). The *NbG6PDH* gene has been negatively correlated with ChiVMV infection (Yang et al. 2022).

Besides the resistance gene loci that restrict viral accumulation, antioxidant enzymes can also defend against ChiVMV infection in pepper. Recent studies have shown that ChiVMV infection can induce the upregulation of specific peroxidase (POD) genes (such as *CaGPX* family members) in pepper. These enzymes alleviate virus‐induced oxidative damage by scavenging reactive oxygen species (ROS) (New et al. 2023; Wang et al. 2023; Zhang et al. 2024). In resistant pepper lines, a significant enhancement of *Prx34* is related to lignin deposition and cell wall strengthening, thereby limiting the spread of the virus (Li et al. 2025). Other antioxidant enzymes such as ascorbate peroxidase (APX) and glutathione peroxidase (GPX) can also work synergistically with superoxide dismutase (SOD) and catalase (CAT) to reduce the level of lipid peroxidation products (such as malondialdehyde [MDA]) and maintain cellular redox homeostasis (Bulle et al. 2024). Virus‐induced gene silencing (VIGS) technology has demonstrated that heterologous expression of specific POD genes (such as *VviCP1*) can significantly enhance plant resistance to viruses (Castro et al. 2023; Lee et al. 2025).

Class III PODs, a key subgroup of plant PODs, are known to participate in critical redox processes by catalysing the oxidation of substrate molecules coupled with the reduction of hydrogen peroxide (Li et al. 2024). A total of 75 class III POD genes have been identified from the pepper genome. These genes are recognised as pivotal regulators of plant growth, development and responses to biotic and abiotic stresses (González‐Gordo et al. 2023). Previous transcriptional analyses revealed that among these, *CaPOD49* was significantly upregulated in the susceptible cultivar Guijiao12 following ChiVMV infection, whereas this expression was not observed in the resistant cultivar Perennial (unpublished data). Despite this correlative evidence, the precise functional role of *CaPOD49* in mediating ChiVMV infection responses remains largely uncharacterised. To address this knowledge gap, we employed multiple experimental approaches comprising phylogenetic analysis, subcellular localisation, VIGS and heterologous expression in yeast to elucidate the biological function of CaPOD49.

## 2 Results

### 2.1 *CaPOD* Family Expression Patterns and Subcellular Localisation of CaPOD49

**FIGURE 1:**
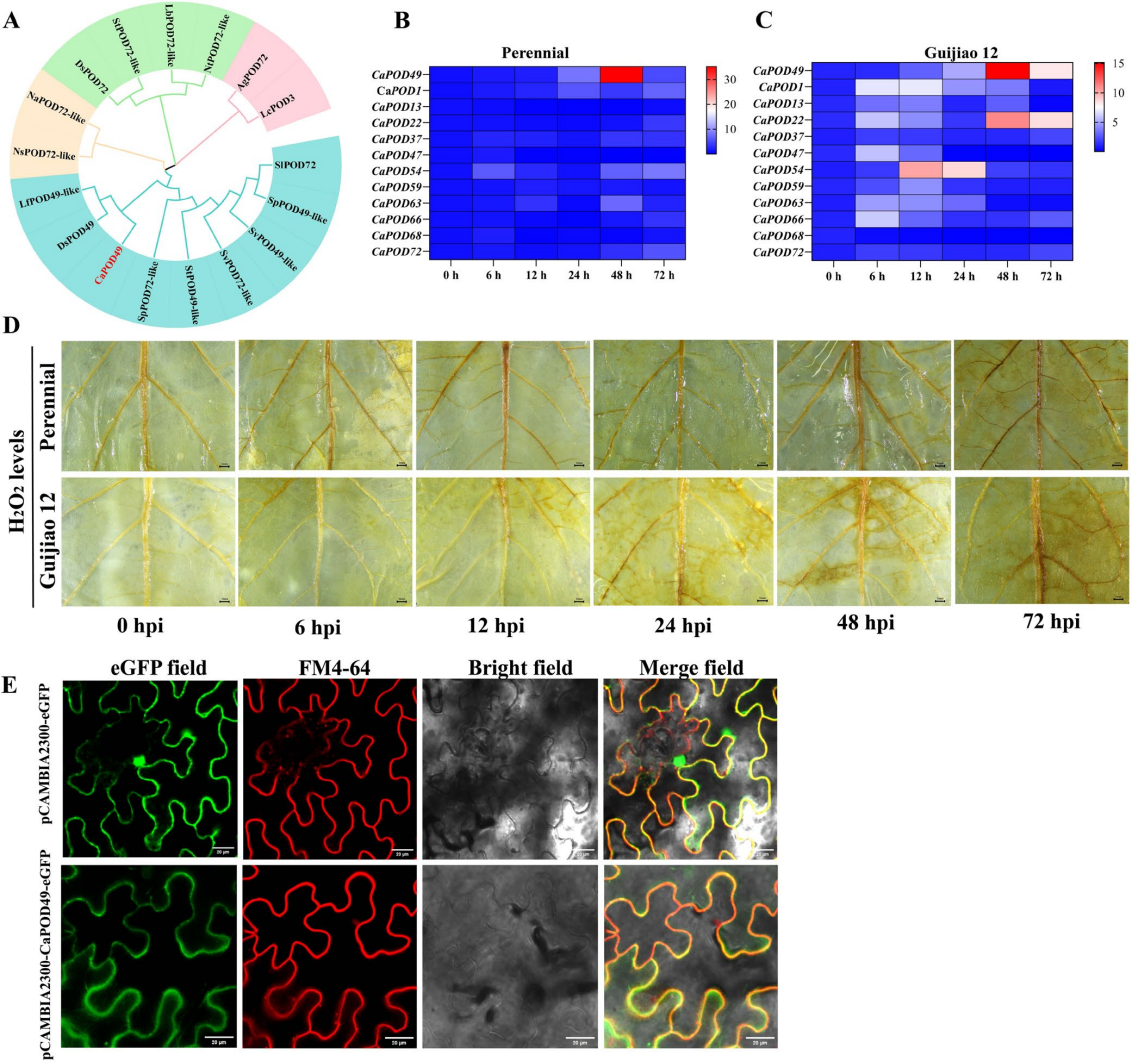
Identification and characterisation of *CaPOD49* in response to chilli veinal mottle virus (ChiVMV) infection. (A) Phylogenetic analysis of pepper peroxidase family. CaPOD49 clusters with a distinct clade of stress‐responsive peroxidases. (B, C) Expression heatmaps showing *CaPOD* gene responses in resistant (Perennial) versus susceptible (Guijiao 12) cultivars from 0 to 72 h post‐infection (hpi). Expression levels are displayed as heatmaps with values normalised to mock‐inoculated controls at each timepoint (0–72 hpi). (D) 3,3′‐diaminobenzidine (DAB) staining reveals differential H_2_O_2_ accumulation between cultivars. (E) CaPOD49‐eGFP localises to the plasma membrane (co‐localisation with FM4‐64). Scale bars: 20 μm.

Previous transcriptome data revealed differential expression of *CaPOD49* between infection in the disease‐sensitive cultivar Guijiao12 and the disease‐resistant pepper cultivar Perennial following ChiVMV infection (unpublished data). Sequence alignment of CaPOD49 revealed conserved class III POD motifs, including heme‐binding histidines and catalytic residues, with variable N‐ and C‐terminal regions (Figure S1). Phylogenetic analysis clustered CaPOD49 into a distinct clade within the plant peroxidase family (Figure 1A). Expression profiling revealed distinct temporal patterns between cultivars following ChiVMV infection. *CaPOD49* was upregulated after 12 h of ChiVMV infection in Guijiao12, and then gradually strengthened from 12 to 48 h, while *CaPOD49* showed delayed expression after 48 h of ChiVMV infection in Perennial (Figure 1B,C). Upregulated expression of *CaPOD1*, *13*, *22*, *47* and *66* was also observed in Guijiao12. H_2_O_2_ accumulation increased progressively with infection severity in both cultivars, with the susceptible cultivar showing consistently higher levels (Figure 1D). CaPOD49‐eGFP localised exclusively to the plasma membrane in *Nicotiana benthamiana* epidermal cells, as confirmed by FM4‐64 co‐staining, whereas 35S‐eGFP showed cytoplasmic and nuclear distribution (Figure 1E).

### 2.2 Oxidative Stress Resistance of Transformed Yeast

**FIGURE 2:**
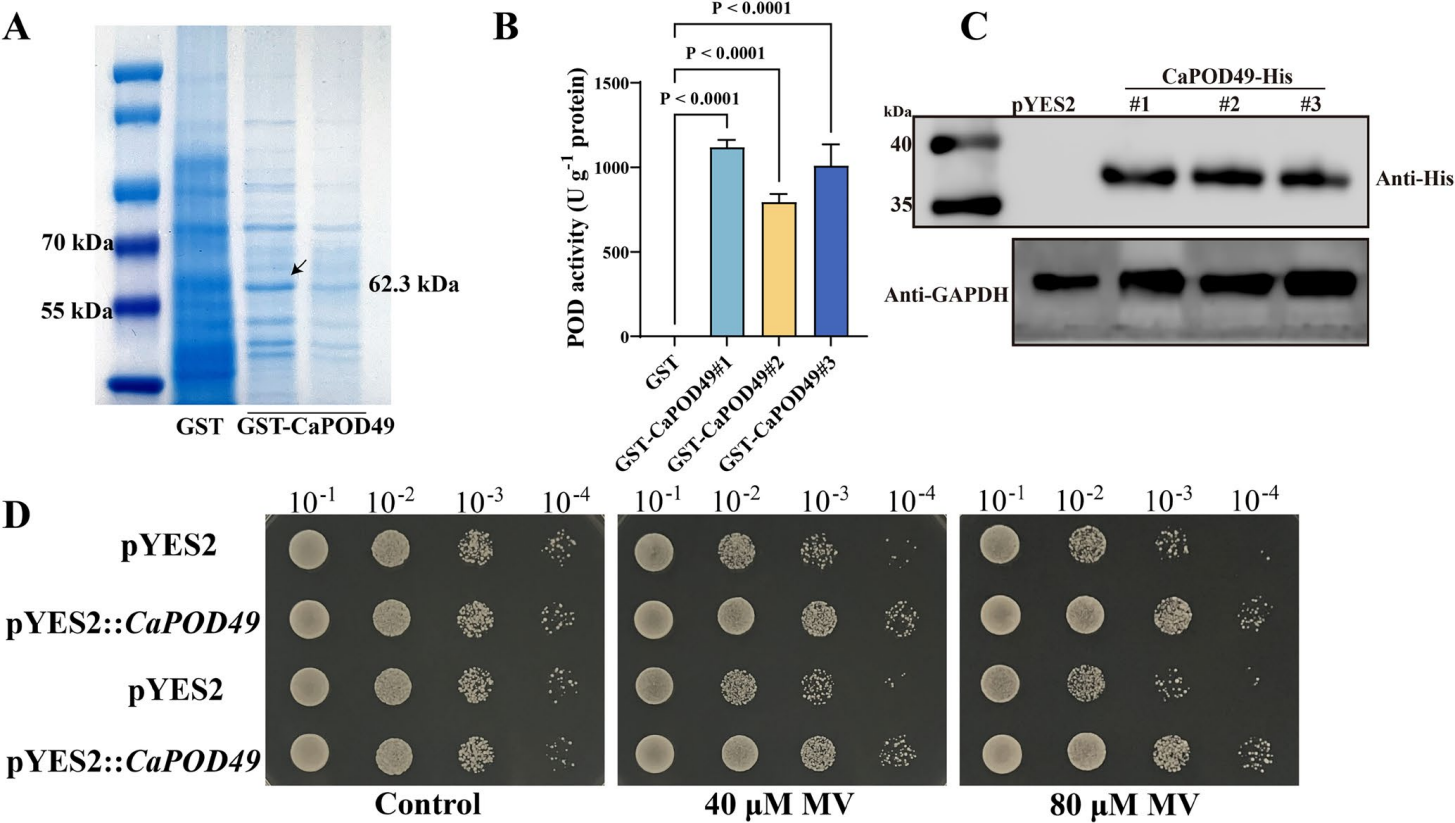
Functional validation of CaPOD49 as an antioxidant enzyme. (A) Purified GST‐CaPOD49 (62.3 kDa) analysed by SDS‐PAGE. Molecular weight markers and glutathione S‐transferase (GST) control (26 kDa) are shown for comparison. (B) Peroxidase activity assay showing GST‐CaPOD49 with significantly higher activity than controls. Data represent mean ± SD (*n* = 3). *p*‐Values indicate statistical significance. (C) Western blot analysis of CaPOD49‐His expression in yeast. Upper panel, CaPOD49‐His protein detected using anti‐His antibody (37.1 kDa); lower panel, GAPDH loading control. pYES2, empty vector control; #1, #2, #3, three independent pYES2‐CaPOD49 transformants. (D) Oxidative stress tolerance assay in *Saccharomyces cerevisiae* . Serial dilutions (10^−1^–10^−4^) of yeast transformants were spotted on selective medium containing 0, 40 or 80 μM methyl viologen (MV).

To investigate the oxidative scavenging function of *CaPOD49*, the coding sequence of *CaPOD49* was cloned into pGEX‐4 T‐1 for bacterial expression and into pYES2 for yeast expression. Optimal induction conditions for GST‐CaPOD49 in *Escherichia coli* (37°C for 4 h) were determined by comparing different temperatures and durations (Figure S2). The recombinant GST‐CaPOD49 protein (62.3 kDa) showed significantly higher peroxidase activity than the GST control (Figure 2A,B). Expression of His‐tagged CaPOD49 in yeast was confirmed by western blot analysis using anti‐His antibody (Figure 2C). When exposed to oxidative stress induced by methyl viologen, yeast expressing *CaPOD49* displayed oxidative stress tolerance compared to empty vector controls, particularly at higher methyl viologen concentrations (Figure 2D). These results validate CaPOD49 as a functional peroxidase capable of reducing oxidative stress.

### 2.3 Effect of *CaPOD49* on ChiVMV Resistance in Chilli Pepper

**FIGURE 3:**
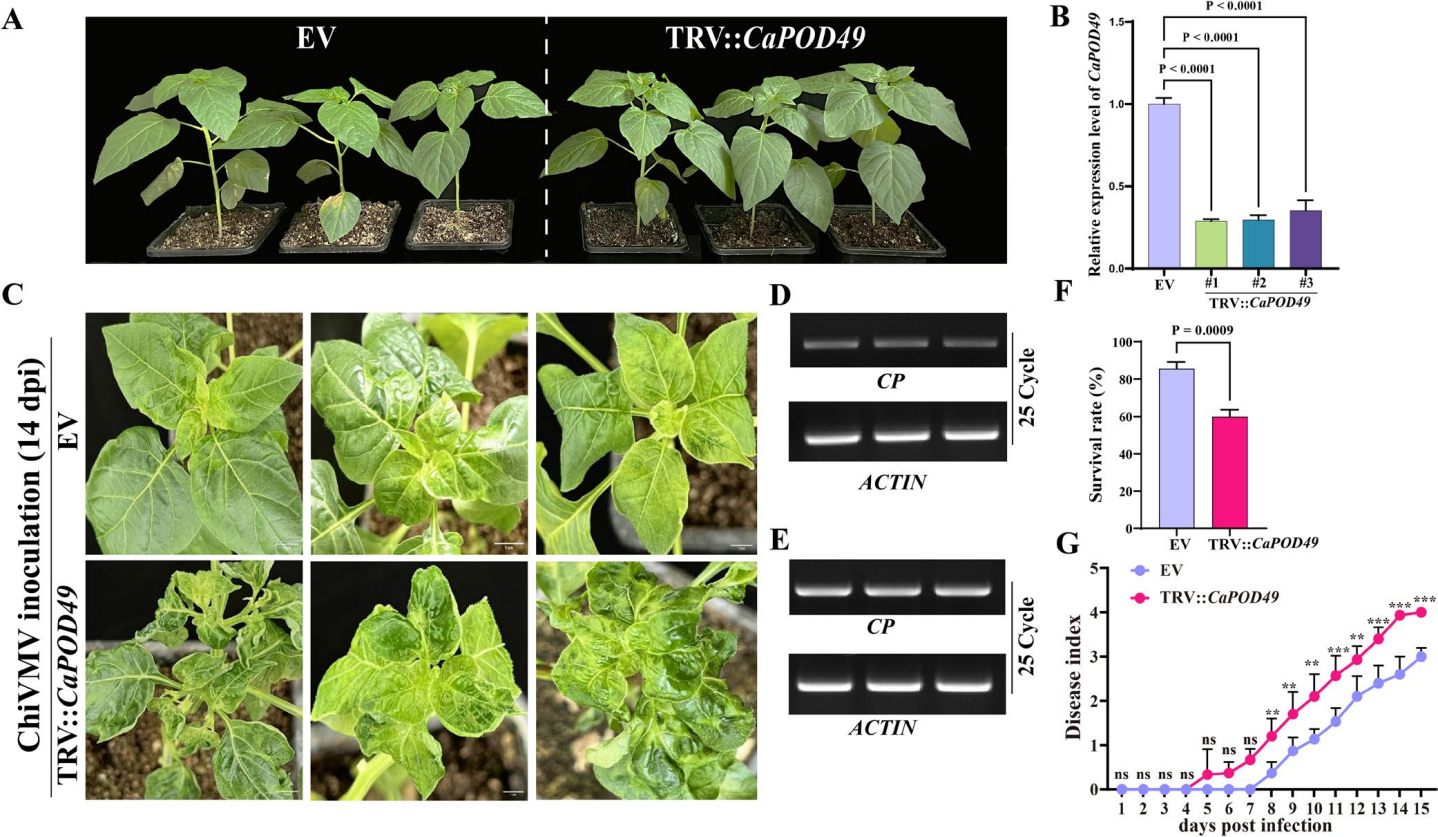
Silencing of *CaPOD49* reduces chilli veinal mottle virus (ChiVMV) resistance in pepper. (A) Phenotypes of empty vector (EV) and TRV‐CaPOD49 plants at 12 days post‐silencing. (B) Relative *CaPOD49* transcript levels in EV and three TRV‐*CaPOD49* lines showing 60%–70% silencing efficiency. Mean ± SEM; one‐way ANOVA. (C) Disease symptoms at 14 days post‐inoculation with ChiVMV. (D, E) Reverse transcription‐PCR of viral *CP* (coat protein) transcripts (25 cycles); *Actin*, internal control. (F) Survival rates post‐ChiVMV infection. TRV‐CaPOD49 plants exhibited significantly reduced survival (Student's *t* test). (G) Disease index over 15 days post‐inoculation. TRV‐CaPOD49 plants displayed elevated disease severity. Values represent mean ± SD (*n* = 18 biological replicates, with at least six plants per treatment in each of three independent experiments). Statistical significance was determined by two‐way ANOVA (**p < 0.01, ***p < 0.001).

To investigate the functional role of the *CaPOD49* gene in viral resistance, VIGS technology was employed to induce target gene silencing. VIGS constructs targeting *CaPOD49* were generated using the pTRV2 vector system and infiltrated into 4‐week‐old chilli pepper seedlings. The *CaPOD49* transcript levels were reduced by 60%–70% at 10 days post‐infiltration (dpi) relative to empty vector (EV) control (Figure 3A,B). Subsequently, *CaPOD49*‐silenced plants, EV controls and mock‐inoculated plants were inoculated with ChiVMV or buffer. Mock‐inoculated plants remained symptom‐free throughout the experiment, confirming that the observed phenotypes were ChiVMV‐specific rather than artefacts of mechanical damage (Figure S3). The *CaPOD49*‐silenced plants exhibited more severe ChiVMV infection symptoms at 15 dpi, including pronounced leaf mottling, wilting and shrinkage (Figure 3C). Disease severity was quantified using a 0–5 disease index scale. A minimum of six plants per treatment group were evaluated across three independent experiments. *CaPOD49*‐silenced plants displayed varying degrees of disease symptoms as early as 5 dpi, whereas EV control plants did not show visible symptoms until 8 dpi. At 15 dpi, *CaPOD49*‐silenced plants showed significantly higher disease index values (DI = 4) compared to EV controls (DI = 3.27) (Figure 3G). No significant difference in plant height was observed between EV and *CaPOD49*‐silenced plants before ChiVMV inoculation (0 dpi). However, *CaPOD49*‐silenced plants showed significantly reduced plant height compared to EV controls at 14 dpi (Figure S4), further confirming the enhanced susceptibility caused by *CaPOD49* silencing. Detection of ChiVMV coat protein (*CP*) gene transcripts was higher in *CaPOD49*‐silenced plants, indicating an enhanced viral accumulation (Figure 3D,E; Figure S5). *CaPOD49*‐silenced plants showed a reduced survival rate (60%) compared to controls (85%) at 20 dpi (Figure 3F).

### 2.4 *CaPOD49* Suppresses the ROS Accumulation After ChiVMV Infection in Chilli Pepper

**FIGURE 4:**
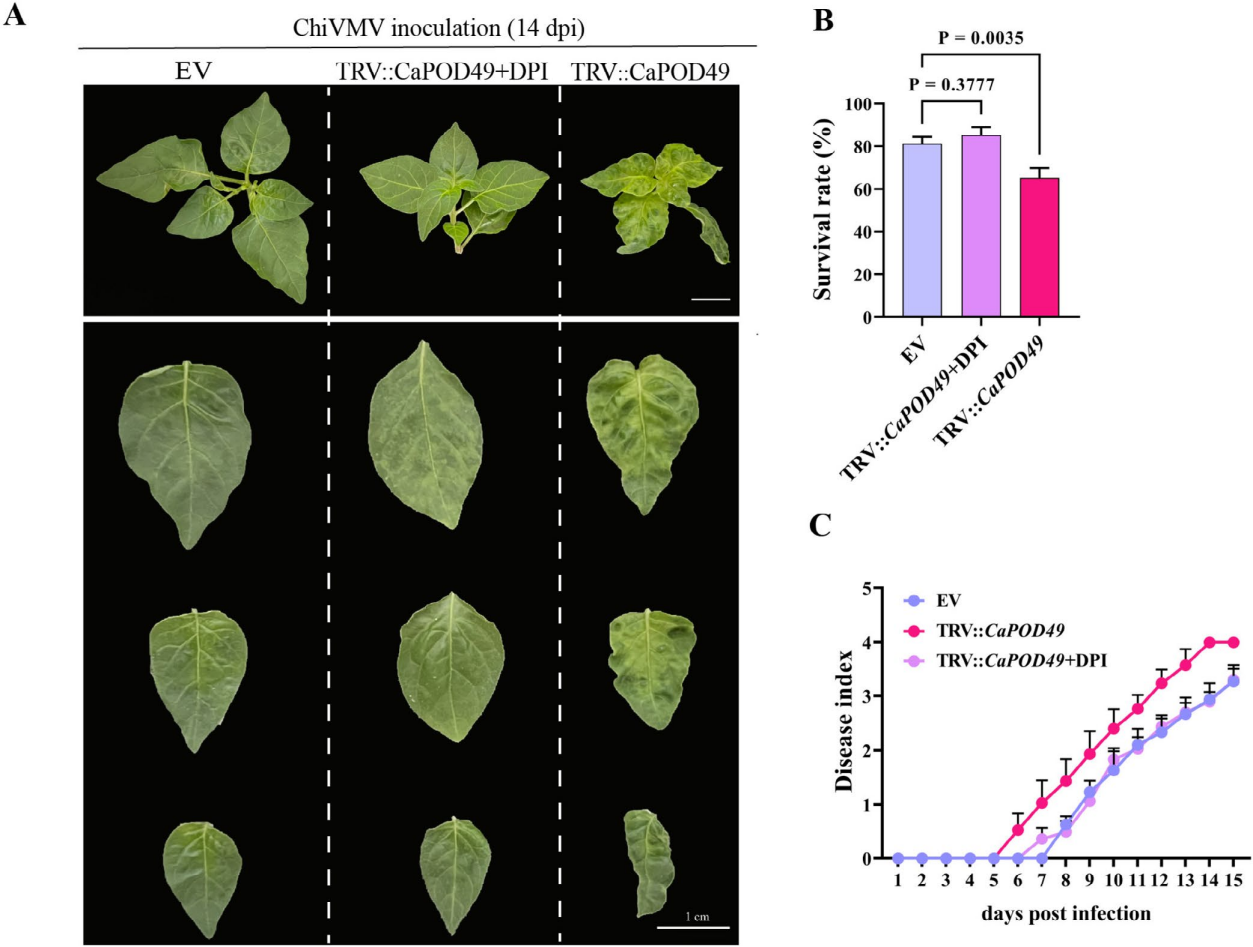
Diphenyleneiodonium (DPI) treatment restores viral resistance in *CaPOD49*‐silenced plants. (A) Representative phenotypes at 14 days post‐inoculation (dpi) with chilli veinal mottle virus (ChiVMV). Plants were treated as follows: Empty vector control (EV), TRV‐CaPOD49 and TRV‐CaPOD49 supplemented with 10 μM DPI. Upper panel shows whole plants. Lower panels display individual leaves. Scale bars: 1 cm. (B) Survival rates post‐ChiVMV infection. DPI treatment significantly enhanced survival in *CaPOD49*‐silenced plants. No significant difference was observed between EV and TRV‐CaPOD49 + DPI groups. One‐way ANOVA. (C) Disease index progression from 1 to 15 dpi. DPI supplementation reduced disease severity in *CaPOD49*‐silenced plants to levels comparable with EV controls.

Given that class III peroxidases regulate intracellular redox homeostasis, we investigated whether *CaPOD49* mediated viral resistance through ROS levels. An NADPH oxidase (NOX) inhibitor (diphenyleneiodonium, DPI) was used to suppress the ROS production during ChiVMV infection. The silencing of *CaPOD49* showed ChiVMV symptoms of mottling and shrinkage in *CaPOD49*‐silenced plants induced by 14 days of ChiVMV infection compared with that of EV plants. When *CaPOD49*‐silenced plants were infected by ChiVMV along with the addition of 10 μM DPI, the ChiVMV infection symptoms were significantly alleviated compared to plants with only *CaPOD49* silencing, showing no obvious difference from the EV plants (Figure 4A and S6). DPI treatment significantly rescued the enhanced susceptibility of *CaPOD49*‐silenced plants to ChiVMV infection. Survival rates at 20 dpi were 81% for EV controls, 65% for *CaPOD49*‐silenced plants and 85% for DPI‐treated silenced plants (Figure 4B). Disease progression analysis confirmed that DPI supplementation delayed symptom initiation and reduced disease severity in *CaPOD49*‐silenced plants to control levels. Additionally, the disease index of the DPI treatment was significantly delayed and lower than that of plants with only *CaPOD49* silencing, which was similar to the EV control (Figure 4C).

**FIGURE 5:**
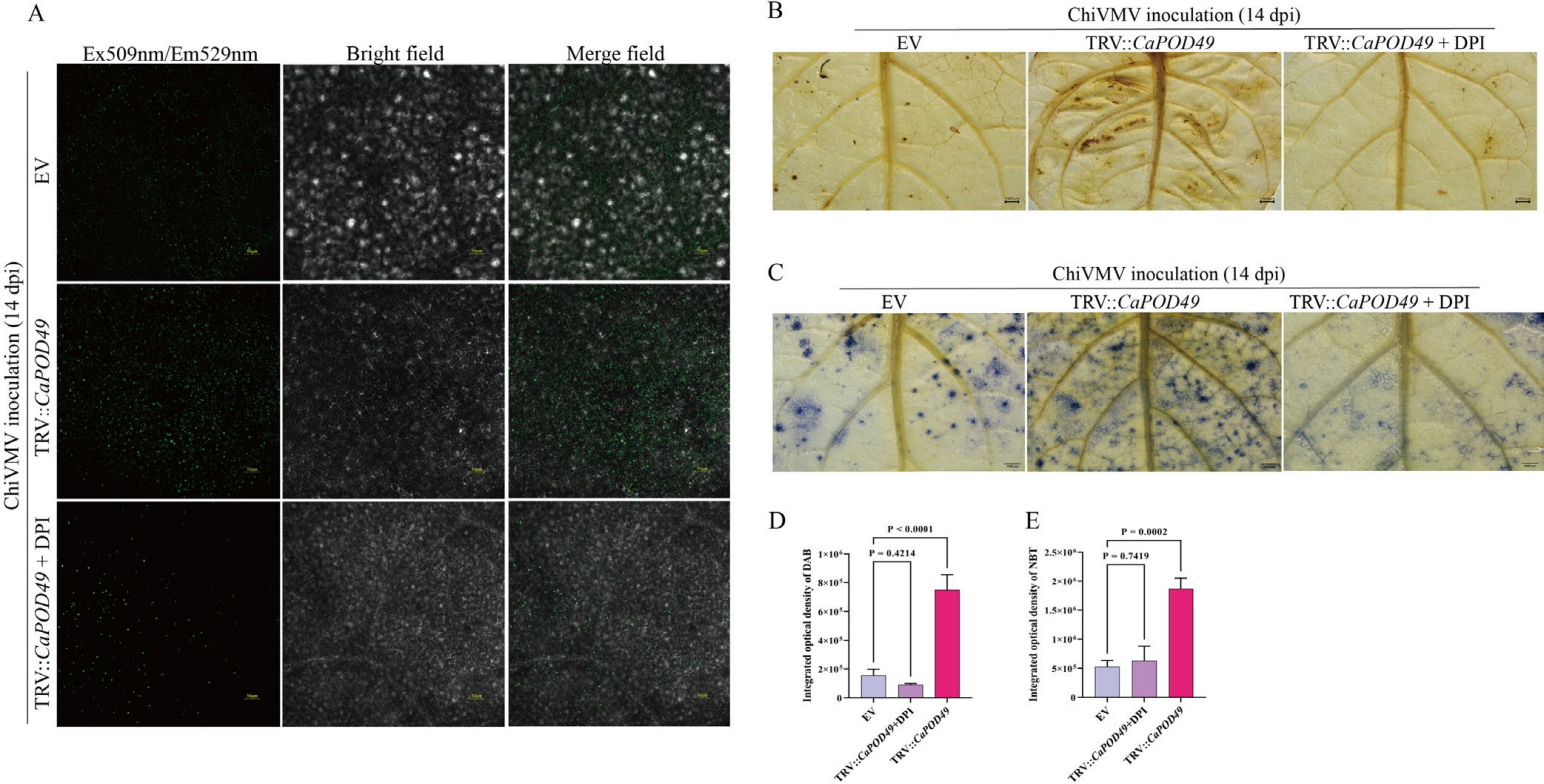
*CaPOD49* silencing enhances reactive oxygen species (ROS) accumulation induced by chilli veinal mottle virus (ChiVMV) infection. (A) ROS detection via 2',7'‐dichlorodihydrofluorescein diacetate (H_2_DCFDA) fluorescence staining at 14 days post‐inoculation (dpi). Empty vector control (EV), TRV‐*CaPOD49* and TRV‐*CaPOD49* supplemented with 10 μM diphenyleneiodonium (DPI). Excitation/emission: 509/529 nm. Scale bars: 100 μm. (B) H_2_O_2_ accumulation visualised by 3,3′‐diaminobenzidine (DAB) staining. (C) O_2_^−^ detection by nitroblue tetrazolium (NBT) staining. Scale bars for (B) and (C): 200 μm. (D, E) Quantification of integrated optical density for DAB and NBT staining, respectively. One‐way ANOVA with Tukey's post hoc test.

To directly assess ROS accumulation in different group plants, ROS fluorescence staining was performed using a 2′,7′‐dichlorodihydrofluorescein diacetate (H_2_DCFDA) probe (Figure 5A). The silencing of *CaPOD49* resulted in more ROS accumulation intracellularly in *CaPOD49*‐silenced plants induced by ChiVMV infection compared to that of EV plants. DPI co‐treatment suppressed the ROS accumulation of ChiVMV infection in *CaPOD49*‐silenced plants (Figure 5A). Detection of H_2_O_2_ content via DAB staining (Figure 5B) and O_2˙_^−^ accumulation via nitroblue tetrazolium (NBT) staining (Figure 5C) revealed that both H_2_O_2_ and O_2˙_^−^ levels were significantly elevated in *CaPOD49*‐silenced plants relative to the EV plants. In contrast, ROS accumulation in the DPI‐treated group was effectively suppressed (Figure 5B,C). The integrated optical density of DAB and NBT revealed a similarly elevated tendency in ROS accumulation among *CaPOD49*‐silenced plants (Figure 5D,E).

### 2.5 CaPOD49 Deficiency Disrupts Cellular Redox Homeostasis and Defence Responses

**FIGURE 6:**
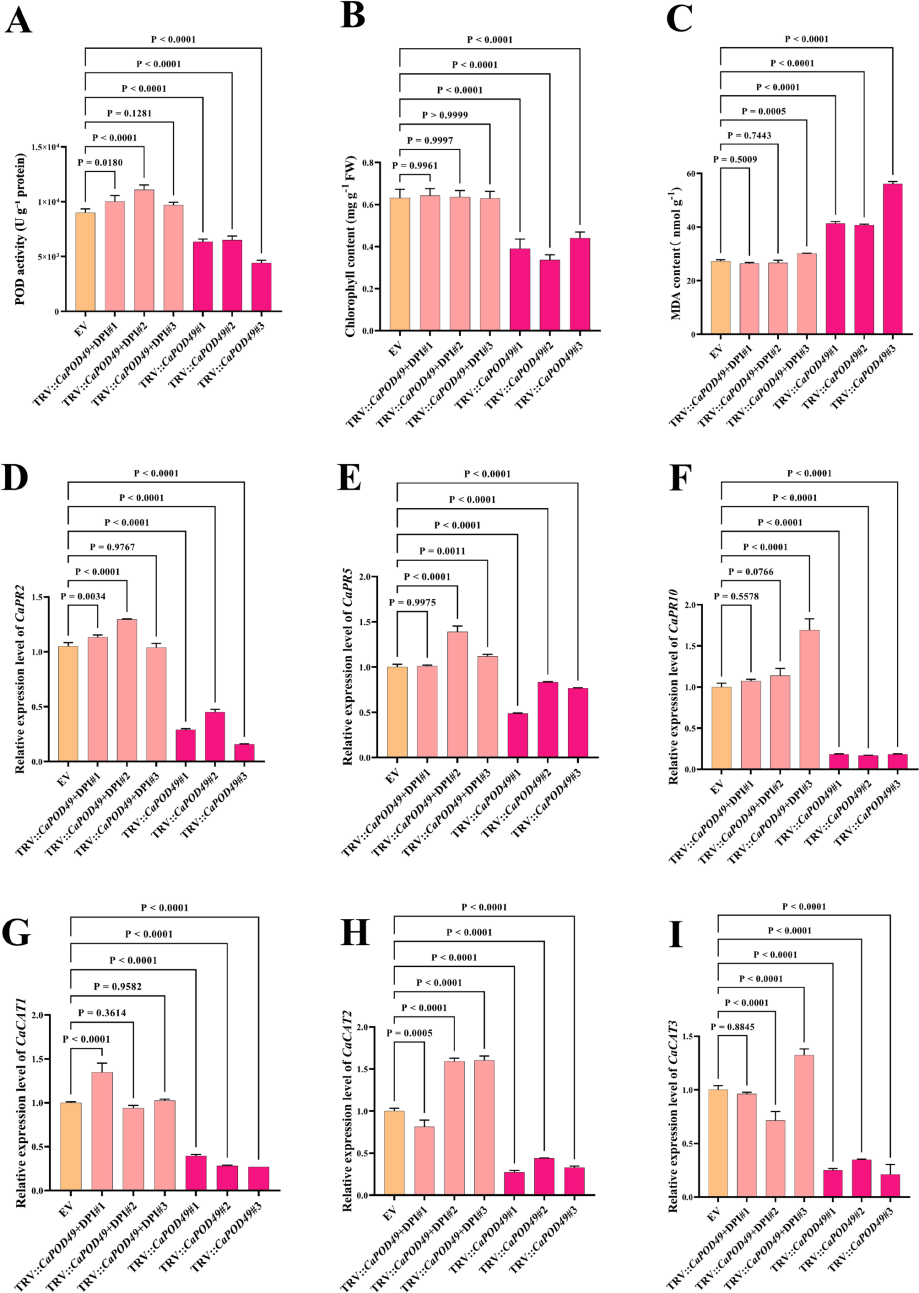
*CaPOD49* silencing impairs antioxidant defence and enhances oxidative damage during chilli veinal mottle virus (ChiVMV) infection. (A) Peroxidase activity in TRV‐*CaPOD49* plants decreased compare to empty vector (EV) plants, with diphenyleneiodonium (DPI) treatment providing recovery. (B) Chlorophyll content was reduced in silenced plants. (C) Malondialdehyde (MDA) accumulation as a marker of membrane damage. (D–F) Defence marker gene expression. *CaPR2* (D), *CaPR5* (E) and *CaPR10* (F) transcripts were suppressed in *CaPOD49*‐silenced plants. (G–I) Catalase gene expression analysis. *CaCAT1* (G), *CaCAT2* (H) and *CaCAT3* (I) showed reduced transcript abundance in silenced plants. DPI treatment (10 μM) partially restores these effects. Values represent mean ± SEM (*n* = 3). Statistical significance determined by one‐way ANOVA.

To elucidate the biochemical basis of enhanced susceptibility in *CaPOD49*‐silenced plants, we analysed MDA content and defence‐related gene expression. *CaPOD49*‐silenced plants showed a decrease of 64% in total peroxidase activity compared to EV controls, confirming functional loss of this enzyme (Figure 6A). Enzyme activities were restored by DPI treatment, suggesting that oxidative stress directly impairs antioxidant enzyme capacity. Chlorophyll content was decreased by approximately 60% in *CaPOD49*‐silenced plants, with DPI treatment providing minimal protection against chlorophyll degradation (Figure 6B). MDA content was increased significantly in silenced plants relative to controls and incompletely rescued by DPI treatment (Figure 6C), revealing significant cellular stress in *CaPOD49*‐silenced plants after ChiVMV infection. Defence gene expression analysis revealed complex regulatory changes. *CaPR2* transcript levels were decreased by 3.5‐fold in *CaPOD49*‐silenced plants, with restoration by DPI treatment (Figure 6D). Similarly, *CaPR5* and *CaPR10* expression were suppressed in silenced plants (Figure 6E,F). Significant reduction in *CaCAT1*, *CaCAT2* and *CaCAT3* gene expression was observed in *CaPOD49*‐silenced plants relative to controls. Catalase gene suppression was alleviated by DPI supplementation in *CaPOD49*‐silenced plants (Figure 6G–I). These expression patterns indicate that *CaPOD49* deficiency disrupts both ROS homeostasis and defence signalling pathways, contributing to enhanced viral susceptibility.

## 3 Discussion

The contrasting expression patterns of *CaPOD49* between resistant and susceptible cultivars reveal complex timing‐dependent defence mechanisms. *CaPOD49* encodes a class III peroxidase typically associated with ROS scavenging. Early *CaPOD49* expression in susceptible Guijiao12 coincided with rapid disease progression, while delayed upregulation in resistant Perennial was likely to be correlated with the effective containment of viral spread. These results suggest that premature ROS scavenging may interfere with early defence signalling, whereas the initiation of appropriately timed peroxidase activity supports sustained immunity (Yang et al. 2015; Lee et al. 2020; Mittler et al. 2022). The plasma membrane localisation of CaPOD49 places it at the critical junction of apoplastic ROS signalling and cellular defence activation (Qi et al. 2017). Among the 11 *POD* family members in chilli pepper, only *CaPOD49* transcripts showed a stronger response to ChiVMV infection in both susceptible and resistant cultivars. *CaPOD49* silencing alone compromised viral resistance, demonstrating functional specialisation in the peroxidase family.

*CaPOD49* gene silencing significantly increased ChiVMV disease severity and reduced plant survival rates (Figure 3D,E), demonstrating the importance of this peroxidase in chilli pepper antiviral defence. *CaPOD49*‐silenced plants exhibited excessive ROS accumulation (Figure 4), elevated lipid peroxidation and suppressed expression of pathogenesis‐related genes (*CaPR2*, *CaPR5*, *CaPR10*), indicating that *CaPOD49* deficiency disrupts normal defence responses against viral infection. The rescue of viral susceptibility by DPI treatment in *CaPOD49*‐silenced plants reveals complex crosstalk between ROS generation and scavenging systems. NADPH oxidases (RBOHs) generate apoplastic superoxide that dismutates to H_2_O_2_, which then serves as substrate for peroxidases in cross‐linking reactions and signalling (Torres and Dangl 2005; Chapman et al. 2019). By inhibiting RBOH activity, DPI prevents the apoplastic H_2_O_2_ accumulation that occurs when *CaPOD49* is absent, thereby avoiding the oxidative damage that facilitates viral spread. Similar studies have indicated that the increased disease susceptibility is correlated with higher H_2_O_2_ and superoxide levels, suggesting that uncontrolled oxidative stress creates favourable conditions for ChiVMV replication and spread (Yang, Qiu, et al. 2020). Without CaPOD49 to metabolise apoplastic H_2_O_2_, the excessive H_2_O_2_ accumulation probably triggers cell death that benefits the virus rather than the host (Wang et al. 2020). Elevated H_2_O_2_ and O_2_^−^ levels in silenced plants indicate multiple ROS sources beyond the eliminating ability of plasma membrane NADPH oxidases. Chloroplastic ROS from photosystem I, mitochondrial electron transport chain leakage and peroxisomal oxidases all contribute to the cellular ROS pool during biotic stress (Wang, Liu, et al. 2024; Wang, Li, and Liang 2024). The partial rescue by DPI suggests that RBOH‐derived ROS specifically drives susceptibility, while other sources may contribute to basal defence. This compartment‐specific ROS function is consistent with findings that ROS location, not total amount, determines stress responses (Considine and Foyer 2021).

The suppression of *CaPR2* and *CaPR5* expression in *CaPOD49*‐silenced plants indicates that ROS homeostasis disruption extends beyond direct oxidative damage to compromise defence signalling networks (Figure 6). PR2 (β‐1,3‐glucanase) and PR5 (thaumatin‐like protein, TLPs) are markers of salicylic acid (SA)‐mediated defence, typically induced during incompatible plant–virus interactions (Kumar et al. 2025). Their downregulation suggests that excessive ROS interferes with SA biosynthesis or signalling, potentially through oxidative modification of NPR1 or TGA transcription factors (Spanu 2015; Withers and Dong 2016). The decline of *CaPR10* expression also in silenced plants may represent a disruption of jasmonic acid (JA)‐dependent defence. This finding contrasts with the classical crosstalk between SA and JA responses, where SA pathway suppression normally enhances JA signalling (Oa et al. 2008; Wees and Van Wees and Pieterse 2013). SA pathway marker genes (*CaPR2*, *CaPR5*) and the JA pathway marker (*CaPR10*) gene were coordinately suppressed, revealing systemic defence failure. Because antiviral ribonuclease activity is provided by PR10 proteins (Park et al. 2004), critical RNA degradation capacity is lost when *PR10* expression is suppressed. This parallel disruption of both hormonal pathways is probably caused by compromised MAPK signalling cascades, through which SA and JA balance is normally regulated in response to ROS signals (Al Mamun et al. 2024). This SA and JA disruption during viral infection could explain the enhanced susceptibility, because effective antiviral defence requires coordinated SA signalling and ROS homeostasis. Therefore, *CaPOD49* silencing causes cascading defence failure. The resulting oxidative stress impairs hormone signalling pathways. Without functional hormone signals, PR proteins cannot be induced. This multistep breakdown ultimately compromises the plant's antiviral immunity.

**FIGURE 7:**
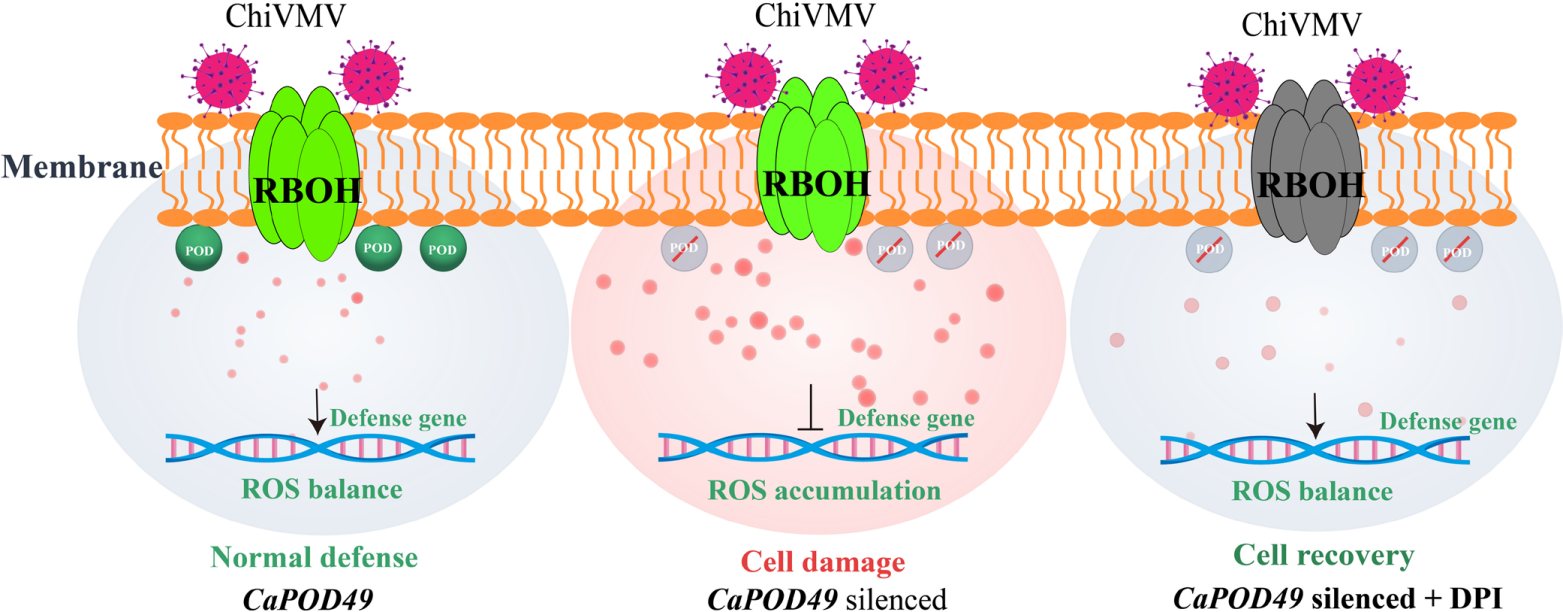
Working model for CaPOD49‐mediated viral resistance through reactive oxygen species (ROS) homeostasis. Left panel: Normal defence response. In plants with functional *CaPOD49*, respiratory burst oxidase homologue (RBOH) generates ROS upon viral recognition. CaPOD49 peroxidase activity (green POD circles) effectively scavenges excess ROS, maintaining redox balance (light blue cytoplasm). This controlled ROS environment enables proper defence signalling, resulting in restricted viral replication and effective antiviral immunity. Centre panel: *CaPOD49* silencing disrupts ROS homeostasis. Loss of CaPOD49 function eliminates critical peroxidase activity (crossed‐out POD symbols), leading to uncontrolled ROS accumulation from RBOH activity (pink cytoplasm indicating oxidative stress). Excessive ROS causes membrane lipid peroxidation, chlorophyll degradation and suppression of defence genes (*CaPR2*, *CaPR5*, *CaPR10*, *CaCAT1*, *CaCAT2, CaCAT3*), resulting in enhanced disease symptoms. Right panel: diphenyleneiodonium (DPI)‐mediated restoration. Application of DPI inhibits RBOH activity (grey inactive RBOH), preventing ROS overproduction in *CaPOD49*‐silenced plants. This pharmacological intervention restores cellular redox balance (light blue cytoplasm), partially rescuing defence responses and reducing viral susceptibility despite the absence of CaPOD49 function. The model demonstrates that maintaining ROS homeostasis, either through CaPOD49 activity or RBOH inhibition, is essential for effective antiviral defence in pepper.

CaPOD49 functions as a critical ROS scavenger whose expression correlates with viral resistance (Figure 7). Higher *CaPOD49* expression in resistant cultivars corresponds with reduced viral accumulation, while lower expression in susceptible cultivars associates with enhanced viral proliferation. This correlation suggests that CaPOD49 levels influence the cellular redox environment during infection. Loss of *CaPOD49* transcription through gene silencing demonstrates its essential role in maintaining redox homeostasis. Silenced plants exhibited excessive ROS accumulation, suppressed defence gene expression (*CaPR2*, *CaPR5*, *CaPR10*) and increased viral susceptibility. These cascading failures indicate that CaPOD49 prevents ROS‐mediated damage to defence signalling networks. DPI treatment partially rescues silenced plants by reducing ROS levels, suggesting that oxidative stress is a primary factor in enhanced susceptibility. The expression of *CaPOD49* in yeast further confirmed its role in preventing oxidative stress. The protective effect of ROS suppression demonstrates that maintaining redox balance is essential for antiviral defence. Both CaPOD49 activity and effective ROS inhibition can achieve this critical balance. These findings establish CaPOD49 as a redox regulator that prevents oxidative damage to defence signalling networks during viral infection.

In conclusion, this study characterises CaPOD49 as an essential redox regulator in chilli pepper antiviral defence against ChiVMV. VIGS‐mediated silencing of *CaPOD49* caused excessive ROS accumulation and systemic suppression of defence markers (*CaPR2*, *CaPR5*, *CaPR10*), resulting in enhanced viral susceptibility. Partial rescue of silenced plants through DPI‐mediated ROS suppression confirmed that oxidative stress drives susceptibility, validating the protective role of redox homeostasis in antiviral immunity. These findings demonstrate that CaPOD49 maintains the cellular redox environment required for functional defence signalling, providing insights for enhancing crop viral resistance through targeted manipulation of ROS regulatory systems.

## 4 Experimental Procedures

### 4.1 Plant Materials and Growth Conditions

Two *C. annuum* cultivars with contrasting ChiVMV susceptibility were used in this study. The susceptible cultivar Guijiao12 and the resistant cultivar Perennial were both obtained from the Guangxi Academy of Agricultural Sciences. Seeds were sown in plastic pots (12 × 12 × 9 cm) containing a mixture of peat, vermiculite and perlite (2:1:1, v/v/v). Plants were cultivated under controlled greenhouse conditions at a daytime temperature of 28°C ± 1°C and a night temperature of 24°C ± 1°C, with a 12 h photoperiod and 70% relative humidity. *Nicotiana benthamiana* plants were grown in the same substrate mixture under similar temperature and humidity conditions but with a 16 h photoperiod. All plants were watered regularly to ensure their normal growth.

### 4.2 Inoculation and Detection of ChiVMV

The ChiVMV isolate was obtained from Guangxi Academy of Agricultural Sciences. To perform mechanical inoculation, infected chilli pepper leaves (1 g) were homogenised in 100 mL of 0.01 M phosphate buffer (pH 7.2–7.8). The surfaces of healthy pepper plant leaves were lightly dusted with carborundum powder. The viral inoculum was gently rubbed onto the leaf surface using a finger, moving from the base towards the tip. Inoculated leaves were thoroughly rinsed with double‐distilled water after 5 min. Mock‐inoculated plants (healthy leaf homogenate in phosphate buffer without virus) served as negative controls to distinguish ChiVMV‐specific effects from mechanical inoculation damage. Total RNA was extracted 12 dpi for reverse transcription and verification of infection status using gene‐specific primers (Table S1). For chemical treatment experiments, DPI stock solution was added to the leaf homogenate from ChiVMV‐infected chilli pepper plants. The mixture was thoroughly vortexed to ensure uniform distribution.

### 4.3 Plasmid Construction

The full‐length coding sequence (CDS) of *CaPOD49* (XM_016705208, 999 bp) was amplified from chilli pepper leaf cDNA via PCR using 2 × Phanta Max Master Mix (Dye Plus, Vazyme). PCR primers were designed with 15–20 nucleotide homologous arms complementary to the linearised vector termini to enable seamless cloning (Table S1). The amplification programme consisted of initial denaturation at 98°C for 3 min; followed by 30 cycles of 98°C for 10 s, 58°C for 15 s and 72°C for 30 s; with a final extension at 72°C for 10 min. The amplified product was verified by agarose gel electrophoresis and matched the predicted CDS length. The purified PCR product was subsequently cloned into the BamHI site of the binary vector p2300‐eGFP using the pEASY‐Basic Seamless Cloning and Assembly Kit (TransGen Biotech) according to the manufacturer's instructions. For heterologous expression in *Saccharomyces cerevisiae* INVSc1 and *E. coli* , the CDS of *CaPOD49* was inserted into the pYES2 and pGEX‐4 T‐1 vectors, respectively. All recombinant constructs were confirmed by Sanger sequencing prior to downstream experiments.

### 4.4 Subcellular Localisation

The p2300‐*CaPOD49*‐eGFP fusion construct and 35S‐eGFP control vector were introduced into *Agrobacterium tumefaciens* LBA4404. Positive transformants were cultured overnight, harvested by centrifugation and resuspended in infiltration buffer (10 mM MES, pH 5.6, 10 mM MgCl_2_, 100 μM acetosyringone) to a final OD_600_ = 0.6. Agrobacteria harbouring the recombinant vector p2300‐*CaPOD49*‐eGFP (or 35S::eGFP) were infiltrated into the leaves of 4‐week‐old *N. benthamiana* plants. After 48 h of dark incubation, leaf segments (1 × 1 cm) were excised and stained with the dye FM4‐64. Fluorescence signals were visualised using an Olympus confocal laser scanning microscope.

### 4.5 VIGS in Chilli Pepper

The *CaPOD49* coding sequence was analysed using the SNG‐VIGS web tool (https://vigs.solgenomics.net/). A 300 bp fragment was selected and amplified by PCR with specific primers (Table S1). This fragment was inserted into the pTRV2 vector using the pEASY‐Basic Seamless Cloning and Assembly Kit (TransGen Biotech). The empty pTRV2 vector was used as a negative control.

Following transformation pTRV1, pTRV2 (empty) and pTRV2‐*CaPOD49* constructs into *A. tumefaciens* GV3101. Bacterial cultures were grown overnight in at 28°C with shaking 200 rpm in Luria Bertani (LB) medium containing rifampicin (25 mg L^−1^), kanamycin (50 mg L^−1^) and acetosyringone (100 μM). Cells were harvested by centrifugation at 4000 *g* for 10 min at 4°C, washed three times with infiltration buffer (10 mM MES, pH 5.6, 10 mM MgCl_2_, 100 μM acetosyringone), and resuspended in infiltration buffer to an OD_600_ = 0.6. Bacterial suspensions were incubated at room temperature in the dark for 2 h before infiltration.

For VIGS experiment, *Agrobacterium* cultures harbouring pTRV1 were mixed in equal volumes with those containing pTRV2 derivatives (empty or *CaPOD49*). The mixed suspensions were infiltrated into the cotyledons of 5‐leaf‐stage chilli pepper seedlings using a needleless syringe. At 10 dpi, newly emerged leaves were collected for total RNA extraction and cDNA synthesis. Gene silencing efficiency was assessed by reverse transcription‐quantitative PCR (RT‐qPCR) using gene‐specific primers (Table S1).

### 4.6 RNA Extraction and Real‐Time RT‐qPCR Analysis

Total RNA was extracted from pepper leaves using the FastPure Universal Plant Total RNA isolation Kit (Vazyme) following the manufacturer's instructions. First‐strand cDNA synthesis was synthesised using SPAPKscript II AII‐in‐one RT SuperMix for RT‐qPCR (SparkJade). Quantitative PCRs were performed in 20 μL volumes containing 10 μL 2 × SYBR Green Master mix (SparkJade), 0.8 μL of each primer (10 μM), 1 μL of cDNA template, and 7.4 μL of nuclease‐free water. Amplification was performed on a SLAN‐965 Real‐Time PCR System (HONGSHI) with the following programme: 95°C for 30 s; followed by 40 cycles of 95°C for 10 s and 60°C for 30 s. Melting curve analysis was performed from 60°C to 90°C with 0.5°C increments to verify amplicon specificity. The chilli pepper *ACTIN* was used as the internal control for normalisation. Relative gene expression levels were calculated using the 2^−ΔΔ*C*t^ method. Each experiment was performed with three biological replicates (independent plants) and three technical replicates per sample.

### 4.7 Physiological Parameter Detection

POD and MDA concentrations were determined as previously described by Chen and Zhang (2018). For enzyme extraction, 0.1 g of leaf tissue was homogenised in extraction buffer. POD activity was determined in a reaction mixture containing 50 μL of extract, 2% guaiacol, 2 mM H_2_O_2_ in 50 mM phosphate buffer (pH 6.0). Activity was determined by monitoring the increases in absorbance at 470 nm at 25°C.

Lipid peroxidation was assessed through MDA quantification, using thiobarbituric acid (TBA) method. Briefly, 0.5 mL extract was combined with 1 mL 0.25% TBA in 10% trichloroacetic acid (TCA), heated at 95°C for 30 min and centrifuged. Absorbances at 532 and 600 nm were recorded, and MDA concentration was calculated using the extinction coefficient of 155 mM^−1^ cm^−1^.

Chlorophyll content was determined according to the method of Arnon (1949) with slight modifications. Fresh tissue (0.1 g) was ground in liquid nitrogen and extracted with 5 mL of 80% acetone at 4°C in the darkness for 24 h. After centrifugation at 10,000 *g* for 10 min at 4°C, absorbance of the supernatant was recorded at 645 nm and 663 nm using an Epoch Microplate Spectrophotometer (BioTek), with 80% acetone as blank. Total chlorophyll content was calculated using the formula: Total chlorophyll (mg/g FW) = [(20.29 × A_645_) + (8.05 × A_663_)] × V/W × 1000, where V is the extract volume (mL) and W is the fresh weight (g) of samples.

### 4.8 DAB and NBT Staining

Hydrogen peroxide (H_2_O_2_) and superoxide (O_2˙_^−^) accumulation were visualised through DAB and NBT staining, respectively, following established protocols. For H_2_O_2_ detection, leaves were immersed in DAB solution (1 mg mL^−1^, pH 5.0) and incubated in the dark at room temperature for 24 h, followed by exposure to light (150 μmol m^−2^ s^−1^) for 2 h to facilitate the oxidation reaction. For O_2˙_^−^ detection, leaves were incubated in NBT solution (0.5 mg mL^−1^ in 50 mM phosphate buffer, pH 7.5) for 24 h in darkness at room temperature. After staining, all samples were decolourised by boiling in 95% ethanol and rehydrated in 4% glycerol for 12 h. The stained tissues were visualised and photographed using a stereomicroscope, and quantitative analysis was performed using Image‐Pro Plus (Media Cybernetics).

### 4.9 Fluorescent Detection of Reactive Oxygen Species

Total ROS accumulation was detected using the fluorescent probe H_2_DCFDA (Shanghai Fushen Biotechnology) following a method modified from Sánchez‐Sanuy et al. (2022). Leaf segments (1 × 1 cm) were vacuum‐infiltrated (−0.08 MPa, 5 min) with 50 μM H_2_DCFDA staining solution for 30 min in darkness at room temperature. Following gentle washing with phosphate‐buffered saline (PBS, 0.1 M, pH 7.4) to remove excess dye, fluorescence was immediately observed using an Olympus confocal laser scanning microscope (excitation: 488 nm; emission: 500–550 nm). A total of 12 leaf segments from four plants (three segments per plant) were analysed for each treatment, and the experiment was repeated three times independently.

### 4.10 Protein Expression and Purification in *E. coli*

The pGEX‐4 T‐*CaPOD49* construct was transformed into *E. coli* Rosetta (DE3) competent cells. A single colony was inoculated into Terrific Broth (TB) medium containing 100 μg mL^−1^ ampicillin and cultured at 37°C until the OD_600_ reached 0.6. Protein expression was induced with 0.5 mM IPTG at 37°C for 4 h. Cells were harvested by centrifugation (4000 *g*, 10 min, 4°C) and resuspended in lysis buffer (TieChui Lysis Buffer, ACE Biotechnology) containing 1 mM PMSF. The lysate was clarified by centrifugation (12,000 *g*, 20 min, 4°C). GST‐CaPOD49 was purified using glutathione resin (BeyoGold). The resin was washed with PBS containing 300 mM NaCl, and bound protein was eluted with 50 mM Tris–HCl (pH 8.0) containing 10 mM reduced glutathione. To remove the residual glutathione that interferes with peroxidase activity, the eluted protein was dialysed overnight at 4°C against 50 mM potassium phosphate buffer (pH 7.0). Protein purity was assessed by SDS‐PAGE and concentration was determined using the Bradford assay. Peroxidase activity of purified protein was measured as described above.

### 4.11 Heterologous Expression in Yeast

The pYES2‐*CaPOD49* construct and the empty vector pYES2 (negative control) were transformed into *S. cerevisiae* INVSc1. Transformants were selected on synthetic dropout medium lacking uracil (SD−U) supplemented with 2% (w/v) glucose at 30°C for 2–3 days. For the oxidative stress tolerance assay, yeast cells were cultured in liquid SD−U medium until they reached the exponential phase. The cells were harvested by centrifugation, washed with sterile water and resuspended to a normalised optical density (OD_600_ = 0.2). A 10‐fold serial dilution series was prepared, and diluted suspensions (10^−1^, 10^−2^, 10^−3^ and 10^−4^) were spotted (5 μL per spot) onto SG−U agar plates (containing 2% galactose) supplemented with 0, 40 or 80 μM methyl viologen (MV). The plates were incubated at 30°C for 3–5 days and photographed.

For western blot analysis, yeast transformants were cultured in liquid SG−U medium (2% galactose) at 30°C for 48 h to induce protein expression. Cells were harvested by centrifugation and washed with sterile water. Cell pellets were resuspended in PBS containing 1 mM PMSF and lysed by sonication on ice. The lysate was clarified by centrifugation (12,000 *g*, 10 min, 4°C), and protein concentration was determined. Equal amounts of total protein were separated by 12% SDS‐PAGE and transferred onto a PVDF membrane. The membrane was blocked with 5% non‐fat milk in Tris‐buffered saline with Tween 20 (TBST) and incubated with a rabbit anti‐His monoclonal antibody (1:3000, Abmart) at 4°C overnight. After washing, the membrane was incubated with horseradish peroxidase (HRP)‐conjugated goat anti‐rabbit secondary antibody (1:5000, Abmart) at room temperature for 1 h. Protein bands were visualised using an enhanced chemiluminescence (ECL, Genestar).

### 4.12 Phylogenetic Analysis

Homologous sequences of *CaPOD49* were retrieved from the National Center for Biotechnology Information (NCBI) database (https://www.ncbi.nlm.nih.gov/) using BLASTN. A neighbour‐joining phylogenetic tree was constructed using the MEGA11 software. The resulting phylogenetic tree was visualised with Evolview (https://www.evolgenius.info/evolview/).

### 4.13 Disease Index Evaluation and Phenotype Quantification

Disease symptoms were monitored and scored daily from 1 to 15 dpi. A single‐blind scoring method was adopted to minimise subjective bias, where plant identities were masked during assessment. The experiment was repeated three times independently, with at least six plants per treatment in each replicate (*n* = 18 biological replicates). For each plant, all leaves were individually scored to calculate a whole‐plant disease index (DI) using a 0–5 scale modified from Krishnareddy et al. (2001) and Chorgasti et al. (2020), where 0 = no visible symptoms; 1 = very mild vein banding or mottling, chlorotic spots on 1%–10% of leaf surface; 2 = clear vein banding and mosaic, slight leaf curling, symptoms on 11%–25% of the leaf area; 3 = severe mottling and banding with obvious leaf distortion and moderate stunting, 26%–40% leaf area affected; 4 = strong vein banding, leaf crinkling and stunting, symptoms affect 41%–60% of the leaf; 5 = very severe leaf deformation, systemic chlorosis or necrosis, plant highly stunted or nearly dead, > 60% of tissue affected. Plant height was measured at 0 and 14 dpi to quantify stunting.

### 4.14 Data Analysis

All experimental data were plotted and statistically analysed using GraphPad Prism 10. Statistical significance was determined using appropriate tests based on the experimental design. For comparisons involving more than two groups, one‐way ANOVA followed by post hoc multiple comparisons tests was performed. For pairwise comparisons between two groups, Student's *t*‐test was applied. *p*‐Values < 0.05 were considered statistically significant. Data are presented as mean ± standard error from at least three independent biological replicates.

## Author Contributions

**Guangqi Wang:** investigation, writing – original draft. **Juanjuan Xu:** investigation, writing – original draft. **Pingchuan Zhu:** investigation (virus infection and statistical analyses). **Zheng Cai:** investigation, writing – original draft. **Youzhi Li:** supervision, project administration. **Mingxia Gong:** investigation (virus infection and statistical analyses). **Bihong Feng:** supervision, project administration. **Risheng Wang:** conceptualization, methodology, writing – review and editing. **Xianwei Fan:** conceptualization, methodology, writing – review and editing. All authors have read and approved the final manuscript.

## Conflicts of Interest

The authors declare no conflicts of interest.

## Supporting information


**Figure S1:** Multiple sequence alignment of CaPOD49 with homologous peroxidases from *Capsicum annuum*.

**Figure S2:** Optimisation of isopropyl β‐d‐1‐thiogalactopyranoside (IPTG) induction conditions for GST‐CaPOD49 expression in *Escherichia coli* .

**Figure S3:** Mock‐inoculated negative control plants.

**Figure S4:** Plant height analysis and phenotypes of EV and TRV‐*CaPOD49* plants after ChiVMV inoculation.

**Figure S5:** Molecular confirmation of viral accumulation after ChiVMV infection in *CaPOD49*‐silenced plants.

**Figure S6:** Comparative viral accumulation in *CaPOD49*‐silenced versus DPI‐treated plants after ChiVMV infection.

**Figure S7:** Uncropped western blot image for Figure 2C.

**Table S1:** Primers used in this study.

## Data Availability

The data that support the findings of this study are available from the corresponding author upon reasonable request. Uncropped images of western blots are provided in the Figure S7.
