# Supplementary material for: Peroxidase Gene CaPOD49 Suppresses Chilli Veinal Mottle Virus Infection and Increases Oxidative Stress Tolerance in Chilli Pepper
Source: Mol Plant Pathol. 2026 Feb 13;27(2):e70222. doi: 10.1111/mpp.70222 (PMC12904604; doi:10.1111/mpp.70222)
Supplement: Supplementary file 1 — Figure S1: Multiple sequence alignment of CaPOD49 with homologous peroxidases from Capsicum annuum. [file MPP-27-e70222-s003.docx]

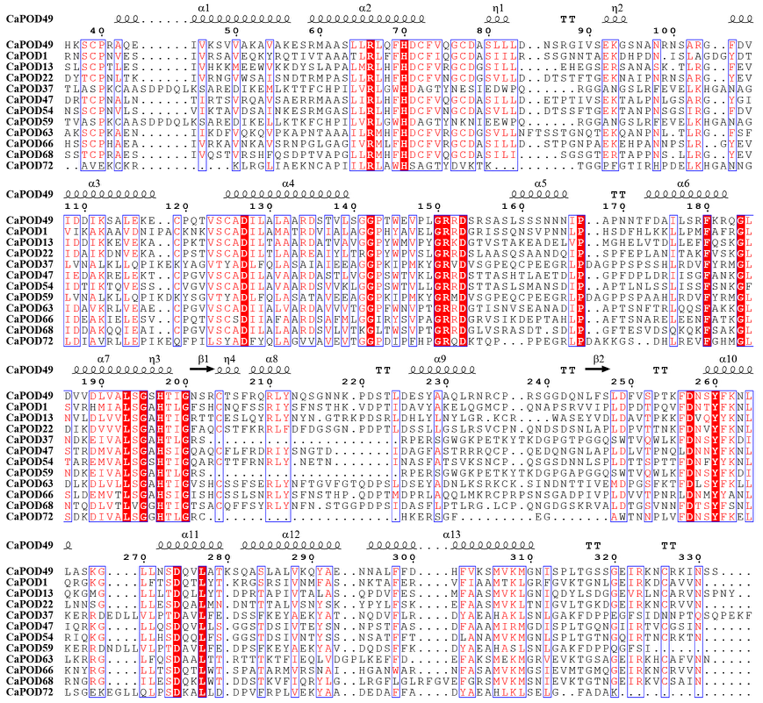


Supplementary figure 1. **Multiple sequence alignment of CaPOD49 with homologous peroxidases from *Capsicum annuum***

Alignment of CaPOD49 amino acid sequence with eleven related peroxidase family members reveals conserved structural motifs and functional domains. Red shading indicates fully conserved residues across all sequences.
