# Supplementary material for: Peroxidase Gene CaPOD49 Suppresses Chilli Veinal Mottle Virus Infection and Increases Oxidative Stress Tolerance in Chilli Pepper
Source: Mol Plant Pathol. 2026 Feb 13;27(2):e70222. doi: 10.1111/mpp.70222 (PMC12904604; doi:10.1111/mpp.70222)
Supplement: Supplementary file 2 — Figure S2: Optimisation of isopropyl β‐d‐1‐thiogalactopyranoside (IPTG) induction conditions for GST‐CaPOD49 expression in Escherichia coli . [file MPP-27-e70222-s004.docx]

**
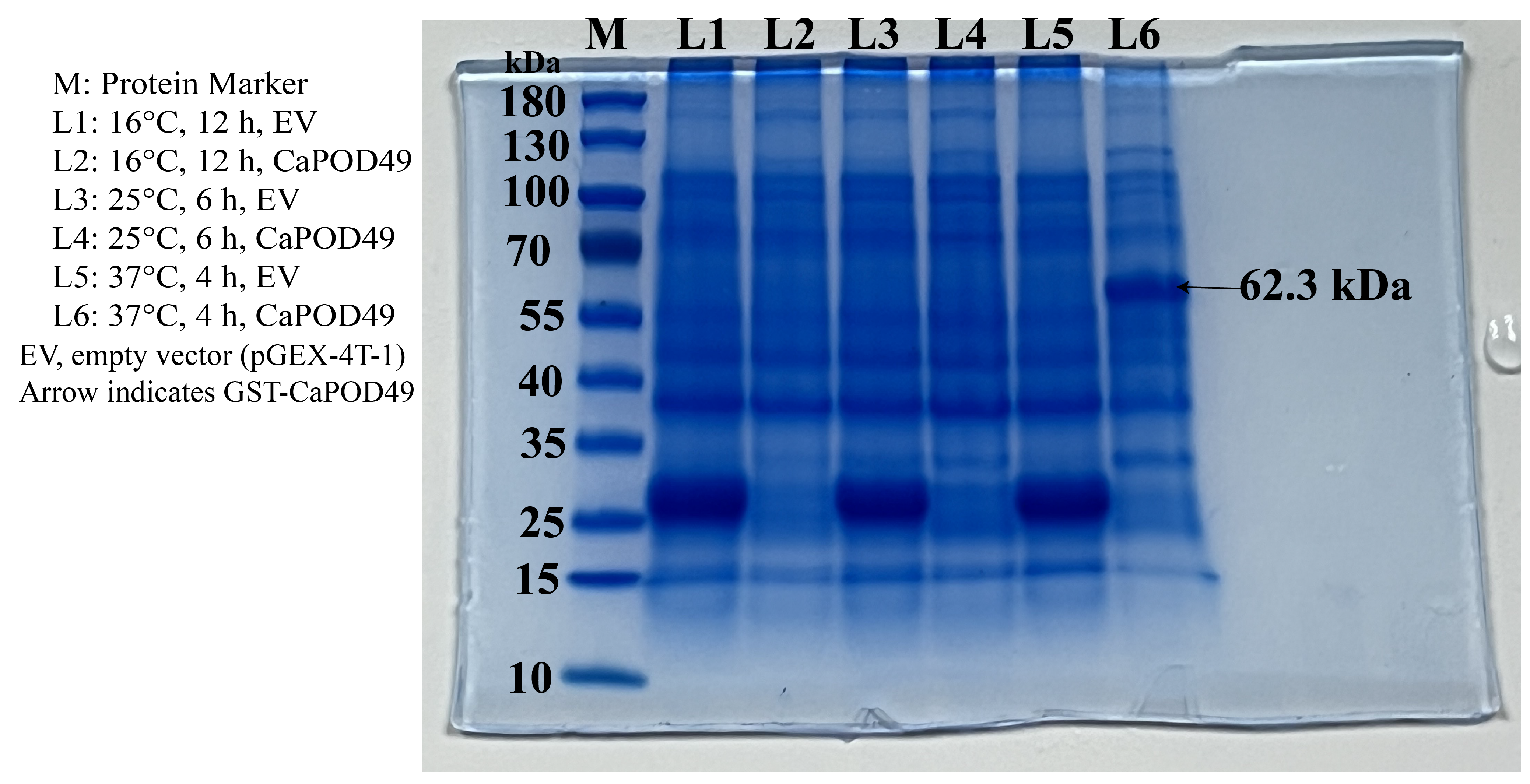
**

Supplementary figure 2. **Optimization of isopropyl β-D-1-thiogalactopyranoside (IPTG) induction conditions for GST-CaPOD49 expression in *E. coli*.**

SDS-PAGE analysis of soluble protein fractions under different induction temperatures and durations. M, protein marker; L1, empty vector (EV) at 16°C for 12 h; L2, pGEX-4T-CaPOD49 at 16°C for 12 h; L3, EV at 25°C for 6 h; L4, pGEX-4T-CaPOD49 at 25°C for 6 h; L5, EV at 37°C for 4 h; L6, pGEX-4T-CaPOD49 at 37°C for 4 h. Arrow indicates the GST-CaPOD49 fusion protein (62.3 kDa). Induction at 37°C for 4 h yielded optimal soluble protein expression.
