# Supplementary material for: Peroxidase Gene CaPOD49 Suppresses Chilli Veinal Mottle Virus Infection and Increases Oxidative Stress Tolerance in Chilli Pepper
Source: Mol Plant Pathol. 2026 Feb 13;27(2):e70222. doi: 10.1111/mpp.70222 (PMC12904604; doi:10.1111/mpp.70222)
Supplement: Supplementary file 3 — Figure S3: Mock‐inoculated negative control plants. [file MPP-27-e70222-s001.docx]

Supplementary figure 3. **Mock-inoculated negative control plants.**

Representative images of chilli pepper plants inoculated with healthy leaf homogenate in phosphate buffer (without ChiVMV) at 14 dpi. Plants showed no visible disease symptoms, confirming that observed phenotypes in ChiVMV-inoculated groups were virus-specific. Scale bar = 1 cm.
