# Supplementary material for: Peroxidase Gene CaPOD49 Suppresses Chilli Veinal Mottle Virus Infection and Increases Oxidative Stress Tolerance in Chilli Pepper
Source: Mol Plant Pathol. 2026 Feb 13;27(2):e70222. doi: 10.1111/mpp.70222 (PMC12904604; doi:10.1111/mpp.70222)
Supplement: Supplementary file 4 — Figure S4: Plant height analysis and phenotypes of EV and TRV‐CaPOD49 plants after ChiVMV inoculation. [file MPP-27-e70222-s007.docx]

Supplementary figure 4. **Plant height analysis and phenotypes of EV and TRV-*CaPOD49* plants after ChiVMV inoculation.**

Left panel, representative images of EV and TRV-*CaPOD49* plants at 14 dpi. Right panel, plant height was measured at 0 dpi (before inoculation) and 14 dpi. No significant difference was observed at 0 dpi (P = 0.4949), whereas TRV-*CaPOD49* plants showed significantly reduced height at 14 dpi (P = 0.0082). Values represent mean ± SD (n = 18). Statistical significance was determined by two-way ANOVA.
