# Supplementary material for: Peroxidase Gene CaPOD49 Suppresses Chilli Veinal Mottle Virus Infection and Increases Oxidative Stress Tolerance in Chilli Pepper
Source: Mol Plant Pathol. 2026 Feb 13;27(2):e70222. doi: 10.1111/mpp.70222 (PMC12904604; doi:10.1111/mpp.70222)
Supplement: Supplementary file 5 — Figure S5: Molecular confirmation of viral accumulation after ChiVMV infection in CaPOD49‐silenced plants. [file MPP-27-e70222-s002.docx]

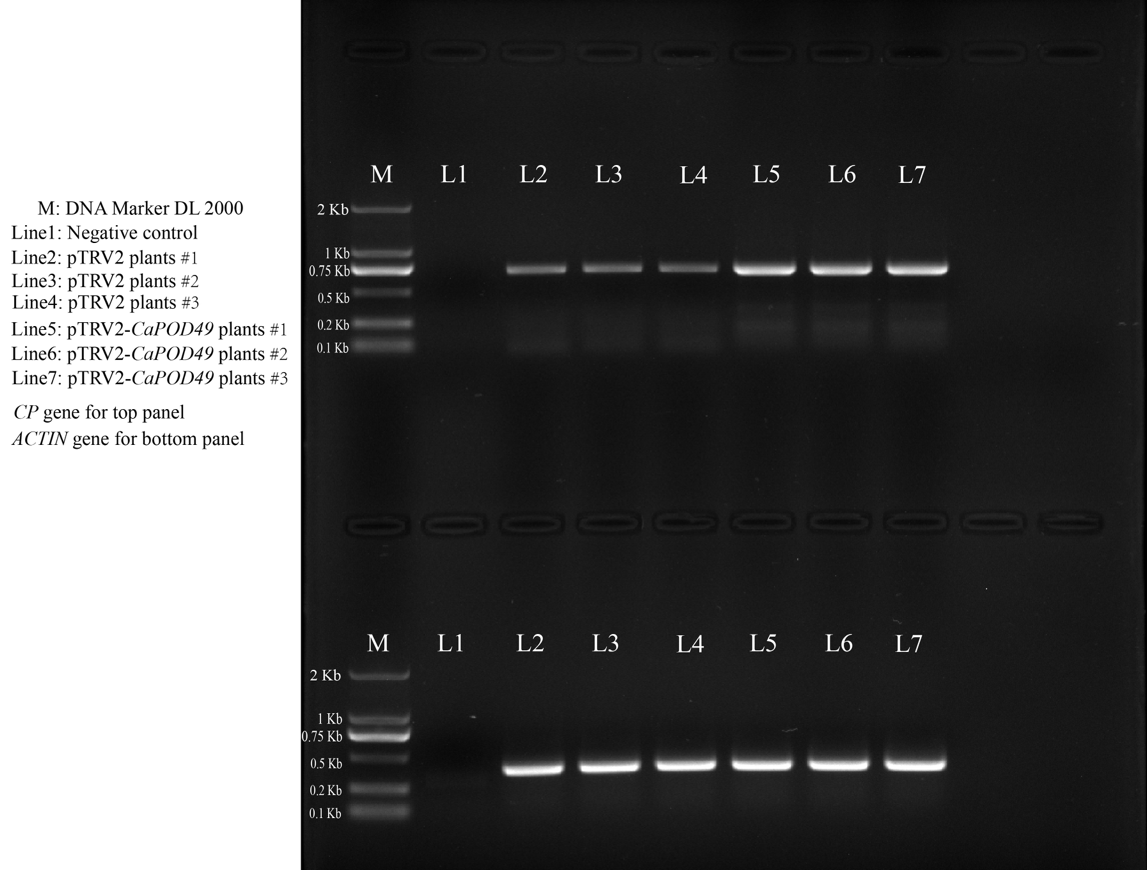


Supplementary figure 5. **Molecular confirmation of viral accumulation after ChiVMV infection in *CaPOD49*-silenced plants**

RT-PCR analysis demonstrating differential viral load between control and *CaPOD49*-silenced plants at 14 dpi. Upper panel: Detection of ChiVMV coat protein (CP) gene transcripts showing enhanced viral accumulation in silenced plants. Lower panel: *ACTIN* expression as internal loading control. M, DNA marker (DL 2000); L1, negative control; L2-L4, pTRV2 empty vector plants (#1-3); L5-L7, pTRV2-*CaPOD49* plants (#1-3). These data correspond to panels D and E from Figure 3
