# Supplementary material for: Peroxidase Gene CaPOD49 Suppresses Chilli Veinal Mottle Virus Infection and Increases Oxidative Stress Tolerance in Chilli Pepper
Source: Mol Plant Pathol. 2026 Feb 13;27(2):e70222. doi: 10.1111/mpp.70222 (PMC12904604; doi:10.1111/mpp.70222)
Supplement: Supplementary file 6 — Figure S6: Comparative viral accumulation in CaPOD49‐silenced versus DPI‐treated plants after ChiVMV infection. [file MPP-27-e70222-s006.docx]

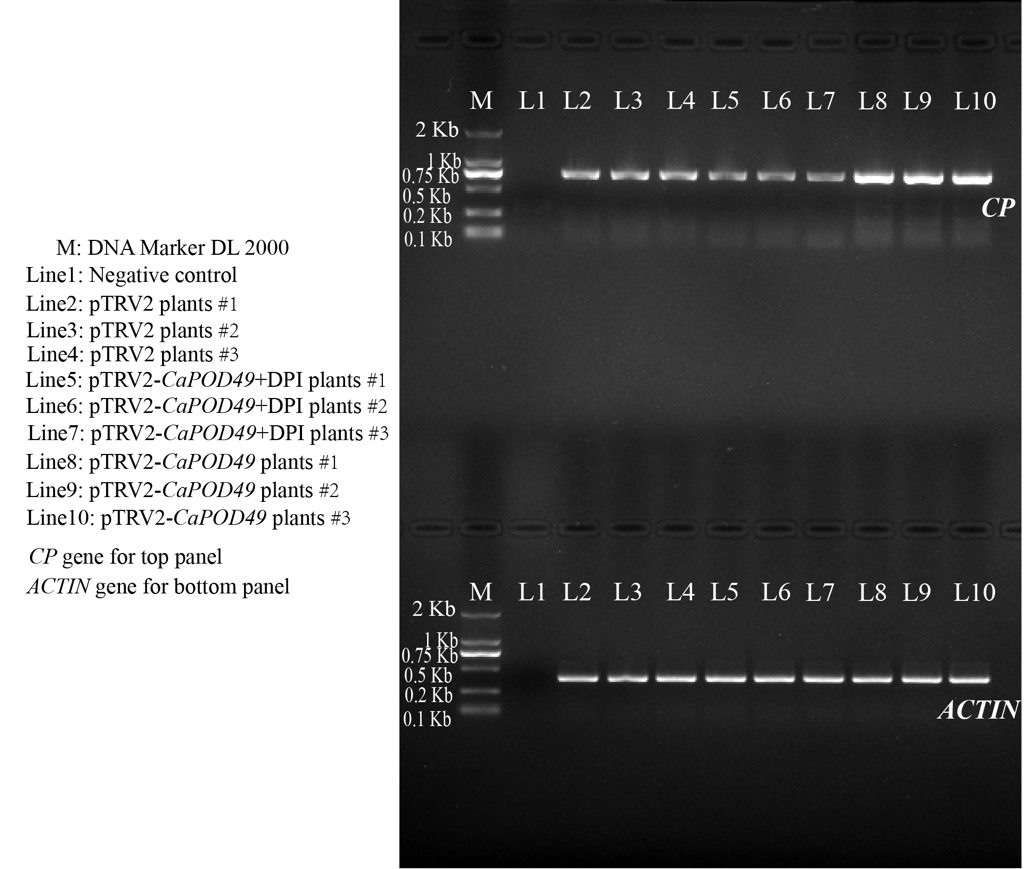


Supplementary figure 6. **Comparative viral accumulation in *CaPOD49*-silenced versus DPI-treated plants after ChiVMV infection**

RT-PCR analysis demonstrating viral load across treatment groups at 14 dpi. Upper panel: ChiVMV coat protein (CP) transcript detection at 25 cycles. Lower panel: *ACTIN* expression as control. M, DNA marker (DL 2000); L1, negative control; L2-L4, pTRV2 empty vector plants (#1-3); L5-L7, pTRV2-*CaPOD49* plants (#1-3); L8-L10, pTRV2-*CaPOD49* + DPI plants (#1-3).
