# Supplementary material for: Peroxidase Gene CaPOD49 Suppresses Chilli Veinal Mottle Virus Infection and Increases Oxidative Stress Tolerance in Chilli Pepper
Source: Mol Plant Pathol. 2026 Feb 13;27(2):e70222. doi: 10.1111/mpp.70222 (PMC12904604; doi:10.1111/mpp.70222)
Supplement: Supplementary file 7 — Figure S7: Uncropped western blot image for Figure 2C. [file MPP-27-e70222-s008.docx]

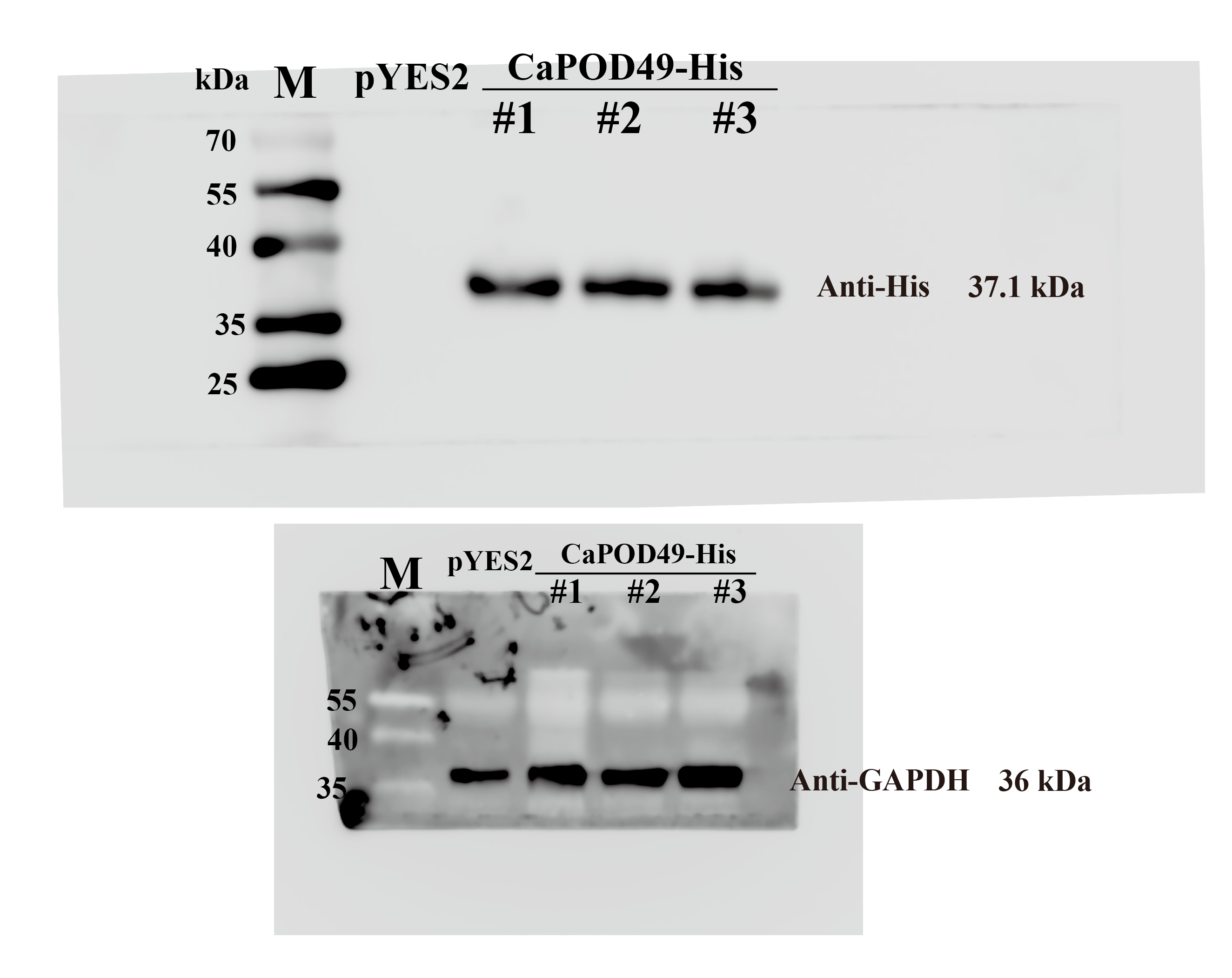


Supplementary figure 7. **Uncropped Western blot image for Fig. 2C.**

Western blot analysis of CaPOD49-His expression in yeast. M, protein marker; pYES2, empty vector control; #1, #2, #3, three independent pYES2-CaPOD49 transformants. Proteins were detected using anti-His antibody. Upper panel, CaPOD49-His protein detected using anti-His antibody (37.1 kDa). Lower panel, GAPDH used as loading control detected using anti-GAPDH antibody (36 kDa).
