# Supplementary material for: Peroxidase Gene CaPOD49 Suppresses Chilli Veinal Mottle Virus Infection and Increases Oxidative Stress Tolerance in Chilli Pepper
Source: Mol Plant Pathol. 2026 Feb 13;27(2):e70222. doi: 10.1111/mpp.70222 (PMC12904604; doi:10.1111/mpp.70222)
Supplement: Supplementary file 8 — Table S1: Primers used in this study. [file MPP-27-e70222-s005.docx]

**Table S1.** Primers used in this study.

| Primer Name | Primer Sequence (5’-3’) | Purpose |
| --- | --- | --- |
| q*CaPOD49*F | GCTCTCCTCGTCTTTGCACC | RT-qPCR analysis |
| q*CaPOD49*R | GCAACAGCCTTGGCAACTAC |  |
| q*CaPOD1*F | CTCCACTTAGTCCTCCTCCTAC |  |
| q*CaPOD1*R | TGCTGCTGTAACAATGGTCTG |  |
| q*CaPOD13*F | AGCAAGAGACGCCACAGTT |  |
| q*CaPOD13* R | GCCTGTATTGTAGAGATTCAC |  |
| q*CaPOD22*F | ATGGCGTCTGGTCTGCTAT |  |
| q*CaPOD22*R | CGATTAGGAATTGCGTTCTTCT |  |
| q*CaPOD37*F | ATTGCTATTGAGGAGGCTGGA |  |
| q*CaPOD37*R | CACTACGCTCTGGTCTGGAT |  |
| q*CaPOD47*F | ATGACCGTACTTGTCCTAATGC |  |
| q*CaPOD47*R | GCAACGCTGTCTTCTCACTAA |  |
| q*CaPOD54*F | GGCTCTCTCAGGTTCTCATACA |  |
| q*CaPOD54*R | GAGGTTGTTGTCACTTCCACTT |  |
| q*CaPOD59*F | CTCCACCACTACTACCAGACTC |  |
| q*CaPOD59*R | CAGGATCAGAGGCAGCACAT |  |
| q*CaPOD63*F | GTGCCAACTGGTAGAAGAGATG |  |
| q*CaPOD63*R | TCGGAGAATGAGGAGCAATGA |  |
| q*CaPOD66*F | CAATTCAACTCACCCGCAAGA |  |
| q*CaPOD66*R | AGTCTGGTCCGAGGTTAATAGC |  |
| q*CaPOD68*F | TTCTGGTTCTGGCACTGAGAG |  |
| q*CaPOD68*R | GCAGCAAGAGCAAGAATGTCA |  |
| q*CaPOD72*F | CGATTGGCGTGGCATTCAG |  |
| q*CaPOD72*R | GTCGGCATAGGATAGGATTGGA |  |
| *CaACTIN*-F | GGAACAGGAATGGTTAAGGC |  |
| *CaACTIN*-R | ATAGCAACATACATGGCAGG |  |
| *CaPR2*-F | CTTGCGGTAGATGCTTGA |  |
| *CaPR2*-R | ACATAGTTGACAGTAAGGTG |  |
| *CaPR5*-F | TGACAAGAGGCAAGTAGTG |  |
| *CaPR5*-R | GACAGAAGGTGAGCAAGTA |  |
| *CaPR10*-F | CAGTGTGATGATGGTGAGA |  |
| *CaPR10*-R | CCTGCTTGGCAATGTCTT |  |
| *CaCAT1*-F | TCCACAAGATTACAGGCATA |  |
| *CaCAT1*-R | AGCGGCAATAGAGTCATAG |  |
| *CaCAT2*-F | CCAATTCCTTCTCGTGTCT |  |
| *CaCAT2*-R | GTATCTGTCTTGCCTGTCA |  |
| *CaCAT3*-F | AGGAGGAGCGAATCATAGT |  |
| *CaCAT3*-R | TTCAATACCAAGCGACCAA |  |
| *CP*-F | CTCAGCCACAGTCTCGTCAGA |  |
| *CP*-R | GCCGTTCAGTGTCCTCTTCCT |  |
| p2300--*CaPOD49*-F | GACAGGGTACCCGGGGATCCATGTCTCGGTCCATGAGCTTCT | Cloning of *CaPOD49* into p2300 vector |
| p2300--*CaPOD49*-R | GTGTCGACTCTAGAGGATCCAGATGAGTTGATCTTCCTGCAATTC |  |
| TRV-*CaPOD49*-F | tcacgcgtctcgaggcccgggATGGCTCGGTCCATGAGCT | Cloning of *CaPOD49* into pTRV2 vector |
| TRV-*CaPOD49*-R | tgtcttcgggacatgcccgggTTCTCCAGTGCAGATTTAATGTCAT |  |
| GST-*CaPOD49*-F | atctggttccgcgtggatccATGGCTCGGTCCATGAGCTT | Cloning of *CaPOD49* into pGEX-4T vector |
| GST-*CaPOD49*-R | cccgggaattccggggatccAGATGAGTTGATCTTCCTGCAATTC |  |
| pYES2-*CaPOD49*-F | ttggtaccgagctcggatccATGGCTCGGTCCATGAGCTT | Cloning of *CaPOD49* into pYES2 vector |
| pYES2-*CaPOD49*-R | cggccgttactagtggatccAGATGAGTTGATCTTCCTGCAATTC |  |
| ChiVMV-F | ATAATTTGTCCCAACCACCTGA | ChiVMV was identified and verified by RT-PCR. |
| ChiVMV-R | CTCACAAGCATTAACACAGAGC |  |
